# Obesity is associated with subclinical hypothyroidism in the presence of thyroid autoantibodies: a cross-sectional study

**DOI:** 10.1186/s12902-022-00981-0

**Published:** 2022-04-08

**Authors:** Yuerong Yan, Mingtong Xu, Muchao Wu, Xiaoyi Wang, Feng Li, Jin Zhang, Lili You, Xiaofang Pan, Weilian Feng, Jiayun Wu, Caixia Chen, Xiaohui Li, Li Yan

**Affiliations:** Department of Endocrinology, Sun Yat-sen Memorial Hospital, Sun Yat-sen University, No. 107 Yanjiang West Road, Guangzhou, 510120 China

**Keywords:** Obesity, Subclinical hypothyroidism, Thyroid autoantibody, BMI, Tg-ab

## Abstract

**Background:**

Both obesity and subclinical hypothyroidism (SCH) have adverse effects on human body, but the relationship between these two conditions remains inconsistent. The presence of thyroid autoantibodies influences thyroid hormone levels, and may further mediate the interaction between obesity and SCH. This study aimed to explore the association among obesity, SCH and thyroid autoantibodies.

**Methods:**

This study was a cross-sectional survey of 2505 subjects. Obesity was defined as a body mass index ≥28 kg/m^2^. Serum concentrations of thyroid hormones, thyroid peroxidase antibody (TPO-Ab) and thyroglobulin antibody (Tg-Ab) were examined. Logistic analysis was used to explore the relation among obesity, SCH and thyroid autoantibodies.

**Results:**

A proportion of 11.54% (289/2505) subjects were obese, and 165 subjects had SCH. The positive rates of thyroid autoantibodies, TPO-Ab and Tg-Ab were 17.64% (442/2505), 11.02% (276/2505) and 14.13% (354/2505), respectively. The proportion of SCH was significantly higher in obese than nonobese subjects among those with positive thyroid autoantibodies [22.41% (13/58) vs. 11.72% (45/384), *p* = 0.025, χ2 test]. Moreover, obesity was significantly associated with SCH in the presence of thyroid autoantibodies after adjusting for confounding factors (OR 2.212, 95% CI 1.103 to 4.433, *p* = 0.025). A higher proportion of subjects with obesity had Tg-Ab positivity [17.99% (52/289) vs. 13.63% (302/2216), *p* = 0.045, χ2 test], and obesity remained significantly associated with Tg-Ab positivity by multiple logistic analysis (OR 1.504, 95% CI 1.077 to 2.101, *p* = 0.017).

**Conclusions:**

Obesity was associated with SCH in the presence of thyroid autoantibodies. Examination of SCH is recommended in obese subjects with thyroid autoantibody positivity.

## Background

At present, obesity is very common worldwide. The global prevalence of obesity increased from 3.2% in 1975 to 10.8% in 2014 in males and from 6.4 to 14.9% in females [1]. Obesity is an important risk factor for many disturbances, such as coronary heart disease, stroke, dyslipidaemia, insulin resistance and type 2 diabetes mellitus [2]. Moreover, obesity is associated with adverse outcomes. Excess weight accounted for 7.2% of all-cause deaths among adults in 2015 [3]. Obesity has become a major public health problem.

Subclinical hypothyroidism (SCH) is defined as mild thyroid failure with elevated thyroid stimulating hormone (TSH) levels and normal free thyroxine (FT4) concentrations. Similar to obesity, SCH is associated with dyslipidaemia, insulin resistance, and atherosclerotic and coronary heart diseases [4, 5]. Moreover, thyroid failure may cause proatherogenic metabolic abnormalities in children with SCH, and thus, it is of great importance to prevent and control SCH [5, 6]. A higher proportion of SCH was observed in obese patients [7, 8]. Obesity may affect thyroid hormones as well, in addition to the role of thyroid hormones in regulating body weight. Hence, identifying the relationship between obesity and SCH could benefit patients by controlling risk factors and improving prognosis. However, several reports failed to find an association between obesity and SCH [9, 10], and their results were inconsistent.

The presence of thyroid autoantibodies is the main aetiology of SCH and influences thyroid hormone levels. Obesity is associated with an increased risk of several autoimmune diseases, including autoimmune thyroiditis [11]. Thus, thyroid autoantibodies may mediate the relationship between obesity and SCH. The current study aimed to investigate the association between obesity and SCH and to further explore the relationship between obesity and SCH in the presence or absence of thyroid autoantibodies.

## Methods

### Subject

This study was a cross-sectional survey of thyroid diseases conducted in Guangzhou, a coastal city in southeastern China. All methods were performed in accordance with the relevant guidelines and regulations. In total, 2720 residents were primarily enrolled. Exclusion criteria included pregnancy, lactation, any medical treatment affecting thyroid function, such as antithyroid drugs, thyroid hormones, glucocorticoids, dopamine, renal insufficiency or any other serious systemic disease. This study was approved by the Medical Ethics Committee of Sun Yat-sen Memorial Hospital of Sun Yat-sen University (AF-SOP-07-1.0-01). Written informed consent was obtained from all subjects before any data or sample collection.

### Assessment

All participants were asked to complete a self-assessment questionnaire that included their date of birth, smoking status and personal or family history of thyroid disease. Overnight fasting blood samples were collected after fasting for more than 10 h and centrifuged at 704×g for 10 min. Subsequently, the serum was decanted for examination. Urine samples were collected between 8:00 am and 10:00 am. All serum and urine samples were stored at − 20 °C and underwent centralized examination within 1 month of collection. The percentage of body fat was measured by a body fat meter (OMRON, HBF-306).

### Laboratory examination

All subjects in this study underwent investigation for serum concentrations of TSH, thyroid peroxidase antibodies (TPO-Ab), and thyroglobulin antibodies (Tg-Ab). If TSH was < 0.27 mU/L, then free thyroxine (FT4) and free triiodothyronine (FT3) were measured in the same sample. If TSH was > 4.20 mU/L, only FT4 was measured. The laboratory reference ranges were as follows: TSH 0.27–4.20 mU/L, FT4 10.30–24.50 pmol/L, FT3 2.30–6.30 pmol/L, TPOAb< 34 IU/L, and TgAb< 50 IU/L. Serum concentrations of thyroid hormones and autoantibodies were examined using chemiluminescence immunoassays on a Cobas 601 analyser (Roche Diagnostics). Urinary iodine concentration was measured by using an As-Ce Catalytic Chromatographer.

### Diagnostic criteria

Subclinical hypothyroidism was defined as normal FT4 and TSH > 4.20 mU/L. Positive TPO-Ab was defined as TPO-Ab ≥34 IU/mL, and positive Tg-Ab was defined as Tg-Ab ≥50 IU/mL. Positive thyroid autoantibodies were defined as TPOAb ≥34 IU/mL and/or TgAb ≥50 IU/mL. Positive TPO-Ab and Tg-Ab were defined as TPOAb ≥34 IU/mL and TgAb ≥50 IU/mL both.

Body mass index (BMI) was calculated by dividing the weight by the square of height (kg/m^2^), and obesity was defined as BMI equal to or greater than 28 kg/m^2^ according to the Guideline for primary care of obesity in China [12].

### Statistical analysis

Data analysis was performed using SPSS version 21.0. Continuous data were reported as the means±standard deviations (SD) or medians (interquartile ranges), while categorical data were reported as percentages. Differences in the continuous data were assessed for significance using nonparametric statistics (Mann–Whitney) or parametric statistics (t test) where appropriate. The chi-squared test or Fisher’s exact test was used to evaluate the differences in the categorical data. Univariate logistic regression analysis was used to identify the potential risk factors associated with subclinical hypothyroidism and thyroid autoantibodies. Factors that were significantly different in univariate logistic regression analysis were then analysed by multiple logistic regression analysis to calculate the odds ratios (ORs) and 95% confidence intervals (95% CIs). In this model, obesity (nonobesity and obesity), subclinical hypothyroidism (no and yes), diabetes mellitus (no and yes), hypertension (no and yes), TPO-Ab (negativity and positivity), Tg-Ab (negativity and positivity), age (< 65 y and ≥ 65 y), sex (male and female) and smoking status (nonsmoking and current smoking) were included as categorical variables. Urinary iodine concentration was included as a continuous variable. Moreover, we utilized a multiple linear regression model to analyse the correlation between BMI and TSH concentration. BMI, TSH concentration, age and urinary iodine concentration were continuous variables, and other factors, including TPO-Ab (negativity and positivity), Tg-Ab (negativity and positivity), sex (male and female) and smoking status (nonsmoking and current smoking), were included as categorical variables. The level of significance was set at 5%.

## Results

### General characteristics of obese and nonobese subjects

Out of the 2720 subjects recruited, 27 subjects who did not complete the survey were excluded. Additionally, another 188 participants with previous thyroid disease were also excluded. Hence, a total of 2505 subjects were included in the final analysis. The mean age was 45.72 ± 15.72 (17 to 88) years, and 55.84% (1399/2505) of the patients were female. A proportion of 11.54% (289/2505) subjects were obese, and 165 subjects had SCH. The rates of positive thyroid autoantibodies, positive TPO-Ab, positive Tg-Ab and both positive these two autoantibodies were 17.64% (442/2505), 11.02% (276/2505), 14.13% (354/2505) and 7.50% (188/2505), respectively. Females had a significantly higher proportion of subclinical hypothyroidism than males [7.72% (108/1399) vs. 5.15% (57/1106), *p* = 0.012]. No significant difference in the ratio of subclinical hypothyroidism was found between subjects ≥65y and < 65y [8.06% (27/335) vs. 6.37% (138/2168), *p* = 0.238]. The general characteristics of obese and nonobese subjects are presented in Table 1.

**Table 1:** General characteristics of obese and nonobese subjects

	Obese subjects (*n* = 289)	Nonobese subjects (*n* = 2216)	*P*
Age(y)	49.07 ± 13.97	45.28 ± 15.89	< 0.001
Age category [n(%)]			0.198
< 65y	243 (84.08%)	1926 (86.91%)	
≥ 65y	46%(15.92%)	290 (13.09%)	
Sex [n(%)]			0.321
Male	135 (46.71%)	971 (43.81%)	
Female	154 (53.29%)	1245 (56.18%)	
BMI (kg/m^2^)	30.39 ± 2.30	22.66 ± 2.77	< 0.001
Waist circumference (cm)	97.18 ± 7.13	80.18 ± 9.31	< 0.001
Hip circumference (cm)	104.85 ± 0.59	93.32 ± 0.13	< 0.001
Percentage of body fat(%)	34.31 ± 6.02	26.71 ± 6.78	< 0.001
Smoking status [n(%)]			0.006
No	209 (72.32%)	1765 (79.65%)	
Yes	80 (27.68%)	451 (20.35%)	
DM [n(%)]			0.035
No	263 (91.00%)	2090 (94.31%)	
Yes	26 (9.00%)	126 (5.69%)	
Hypertension [n(%)]			< 0.001
No	249 (86.16%)	2067 (93.28%)	
Yes	40 (13.84%)	149 (6.72%)	
Urinary iodine concentration (μg/L)	137.00 (94.35–194.50)	128.00 (87.05–181.38)	0.028
Subclinical hypothyroidism [n(%)]			0.045
Yes	27 (9.34%)	138 (6.23%)	
No	262 (90.66%)	2078 (93.77%)	
Positive TPO-Ab and/or Tg-Ab [n(%)]			0.250
Yes	58 (20.07%)	384 (17.33%)	
No	231 (79.93%)	1832 (82.67%)	
Positive TPO-Ab and Tg-Ab [n(%)]			0.032
Yes	31 (10.73%)	157 (7.08%)	
No	258 (89.27%)	2059 (92.92%)	
Positive Tg-Ab [n(%)]			0.045
Yes	52 (17.99%)	302 (13.63%)	
No	237 (82.01%)	1914 (86.37%)	
Positive TPO-Ab [n(%)]			0.303
Yes	37 (12.80%)	239 (10.79%)	
No	252 (87.20%)	1977 (89.21%)	
Serum TSH level (mIU/L)	1.82 (1.25,2.64)	1.97 (1.24,2.70)	0.261

Age, BMI, waist circumference and percentage of body fat were expressed as the means±standard deviations; urinary iodine concentration and serum TSH level was expressed as medians with interquartile ranges; age category, sex, smoking status, DM, hypertension, subclinical hypothyroidism, thyroid autoantibodies, Tg-Ab and TPO-Ab were presented as n (%)

The t test was used for age, BMI, waist circumference and percentage of body fat; the Mann–Whitney U test was adopted for urinary iodine concentration and serum TSH level; the χ2 test was used for age category, sex, smoking status, DM, hypertension, subclinical hypothyroidism, thyroid autoantibodies, Tg-Ab and TPO-Ab. *P* < 0.05 was considered significant

*BMI* Body mass index, *DM* Diabetes mellitus, *TPO-Ab* Thyroid peroxidase antibody, *Tg-Ab* Thyroglobulin antibody, *TSH* Thyroid stimulating hormone

### Obesity and subclinical hypothyroidism

Although univariate logistic analysis found that obesity was associated with SCH among the total subjects, no significant association between obesity and SCH was found in the multiple logistic analysis, as shown in Table 2.

**Table 2:** Risk factors of subclinical hypothyroidism among total subjects, subjects with positive thyroid autoantibodies, and subjects with negative autoantibodies

	Total subjects		Subjects with positive thyroid autoantibodies		Subjects with negative autoantibodies	
	OR(95%CI)	*P*	OR(95%CI)	*P*	OR(95%CI)	*P*
Univariate logistic analysis						
Sex	1.540 (1.106,2.144)	0.011	1.790 (0.847,3.780)	0.127	1.170 (0.791,1.730)	0.431
Age	1.290 (0.839,1.981)	0.246	1.904 (0.489,2.445)	0.827	1.413 (0.846,2.360)	0.186
Smoking	0.250 (0.135,0.464)	< 0.001	0.391 (0.118,1.300)	0.126	0.253 (0.122,0.525)	< 0.001
Obesity	1.552 (1.007,2.390)	0.046	2.176 (1.090,4.344)	0.027	1.206 (0.676,2.153)	0.526
Diabete mellitus	0.973 (0.502,1.887)	0.936	3.316 (0.437,25.132)	0.246	0.694 (0.342,1.407)	0.311
Hypertension	0.950 (0.664,1.358)	0.778	0.967 (0.509,1.839)	0.919	0.936 (0.604,1.450)	0.766
Urinary iodine concentration	1.001 (1.000,1.001)	0.061	1.000 (0.999,1.002)	0.398	1.001 (1.000,1.001)	0.094
TPO-Ab	3.187 (2.195,4.628)	< 0.001	–	–	–	–
Tg-Ab	3.206 (2.262,4.542)	< 0.001	–	–	–	–
Multiple logistic analysis						
Sex	0.820 (0.567,1.185)	0.291	1.857 (0.872,3.958)	0.109	–	–
Age	1.277 (0.823,1.982)	0.275	1.155 (0.509,2.621)	0.730	–	–
Smoking	0.248 (0.127,0.482)	< 0.001	–	–	–	–
Obesity	1.542 (0.988,2.407)	0.057	2.212 (1.103,4.433)	0.025	–	–
TPO-Ab	1.924 (1.190,3.110)	0.008	–	–	–	–
Tg-Ab	2.053 (1.303,3.236)	0.002	–	–	–	–

Univariate logistic regression, when adjusted for sex (male and female), age (<65y and ≥ 65y), smoking status (non-smoking and smoking), obesity (no and yes), diabetes mellitus (no and yes), hypertension (no and yes), urinary iodine concentration (continuous variable), TPO-Ab (negativity and positivity) and Tg-Ab (negativity and positivity) was used for identify the associated risk factors for subclinical hypothyroidism among the total subjects, subjects with positive thyroid autoantibodies, and subjects with negative autoantibodies, respectively. Factors that differed in the univariate logistic analysis were further analysed in the multiple logistic analysis. P < 0.05 was considered significant

*TPO-Ab* Thyroid peroxidase antibody, *Tg-A*b Thyroglobulin antibody

When we divided all subjects into two subgroups (442 subjects with positive thyroid autoantibodies and 2063 subjects with negative thyroid autoantibodies), the results showed that only among subjects with positive thyroid autoantibodies obese subjects had a significantly higher ratio of SCH than nonobese subjects [22.41% (13/58) vs. 11.72% (45/384), *p* = 0.025, χ2 test]. Multiple logistic analysis showed that obesity was associated with subclinical hypothyroidism among these subjects (OR 2.212, 95% CI 1.103 to 4.433, *p* = 0.025, Table 2). However, no significant difference in the proportion of SCH [obese 6.06% (14/231) vs. nonobese 5.08% (93/1832), *p* = 0.525, χ2 test] or the association between obesity and SCH (OR 2.176, 95% CI 1.090 to 4.344, *p* = 0.027, univariate logistic analysis) was found among subjects with negative thyroid autoantibodies.

### TSH concentration, BMI and thyroid autoantibodies

The median TSH concentration of all 2505 subjects was 1.83 (1.25–2.65) mU/L. As shown in Table 3, subjects with positive thyroid autoantibodies had a significantly higher TSH concentration than subjects with negative thyroid autoantibodies. In the linear regression analysis, BMI was significantly correlated with TSH concentration, regardless of whether subjects were positive or negative for thyroid autoantibodies (Table 4).

**Table 3:** Serum TSH concentration in subjects with or without thyroid autoantibodies

TSH	TPO-Ab	Tg-Ab	TPO-Ab and Tg-Ab	TPO-Ab and/or Tg-Ab
Ab(+)	2.10 (1.33,3.37)	2.07 (1.33,3.31)	2.44 (1.49,4.07)^a^	1.98 (1.25,3.14)^b^
Ab(−)	1.81 (1.24,2.58)	1.81 (1.24,2.58)	1.81 (1.24,2.58)	1.81 (1.25,2.58)
P	< 0.001	< 0.001	< 0.001	0.008

^a^both TPO-Ab and Tg-Ab positive; ^b^either TPO-Ab or Tg-Ab positive

Serum TSH level was expressed as medians with interquartile ranges

Mann–Whitney U test was adopted for serum TSH level. *P* < 0.05 was considered significant

*TPO-Ab* Thyroid peroxidase antibody, *Tg-Ab* Thyroglobulin antibody, *TSH* Thyroid stimulating hormone, *Ab* Antibody

**Table 4:** Correlations of body mass index and thyroid stimulating hormone among total subjects, subjects with positive thyroid autoantibodies, and subjects with negative autoantibodies

	Total subjects		Subjects with positive thyroid autoantibodies		Subjects with negative autoantibodies	
	r(95%CI)	*P*	r(95%CI)	*P*	r(95%CI)	*P*
Sex	−0.201(− 0.556,0.153)	0.266	− 0.342(−2.556,1.871)	0.761	− 0.146(− 0.293, 0.002)	0.053
Age	− 0.009(− 0.018, 0.001),	0.072	− 0.024(− 0.078,0.030)	0.379	−0.005(−.009,0.000)	0.016
Smoking	−0.559(− 0.983,-0.134)	0.010	−1.736(−4.693, 1.221)	0.249	−0.415(− 0.588,-0.241)	< 0.001
BMI	0.063 (0.022,0.104)	0.003	0.211 (0.007, 0.415)	0.043	0.024 (0.006,0.042)	0.008
Urinary iodine concentration	0.000 (0.000,0.001)	0.071	−0.001(−0.005,0.003)	0.578	0.000 (0.000,0.001)	0.049
TPO-Ab	1.300 (0.747, 1.853)	< 0.001	–	–	–	–
Tg-Ab	0.740 (0.237,1.243)	0.004	–	–	–	–

Multiple liner regression, when adjusted for sex (male and female), age (continuous variable), smoking status (non-smoking and smoking), BMI (continuous variable), urinary iodine concentration (continuous variable), TPO-Ab (negativity and positivity) and Tg-Ab (negativity and positivity) was used for identify the correlation of TSH among the total subjects; Multiple liner regression, when adjusted for sex (male and female), age (continuous variable), smoking status (non-smoking and smoking), BMI (continuous variable) and urinary iodine concentration (continuous variable) was used for identify the correlation of TSH among subjects with positive thyroid autoantibodies, and subjects with negative autoantibodies, respectively. *P* < 0.05 was considered significant

*BMI* Body mass index, *TPO-Ab* Thyroid peroxidase antibody, *Tg-Ab* thyroglobulin antibody

### Obesity and thyroid autoantibodies

Among the 2505 subjects, the rates of TPO-Ab and Tg-Ab positivity were 11.02% (276/2505) and 14.13% (354/2505), respectively. Obesity was significantly associated with Tg-Ab both in the univariate and multiple logistic analyses (Table 5). No significant associations between obesity and thyroid autoantibodies (OR 1.198, 95% CI 0.880 to 1.630, *p* = 0.251) or TPO-Ab (OR 1.215, 95% CI 0.839 to 1.759, *p* = 0.304) were found in the univariate analysis.

**Table 5:** Risk factors of positive thyroid autoantibodies, positive TPO-Ab and positive Tg-Ab

	Model 1		Model 2		Model 3	
	OR(95%CI)	*P*	OR(95%CI)	*P*	OR(95%CI)	*P*
Univariate logistic analysis						
Sex	3.069 (2.424,3.887)	< 0.001	2.402 (1.815,3.178)	< 0.001	3.845 (2.917,5.068)	< 0.001
Age	0.950 (0.700,1.289)	0.740	1.038 (0.721,1.492)	0.843	0.853 (0.604,1.204)	0.365
Smoking	0.420 (0.307,0.573)	< 0.001	0.581 (0.408,0.827)	0.003	0.293 (0.198,0.434)	< 0.001
Obesity	1.198 (0.880,1.630)	0.251	1.215 (0.839,1.759)	0.304	1.391 (1.006,1.922)	0.046
Diabetes Mellitus	1.237 (0.777,1.970)	0.370	0.889 (0.534,1.481)	0.652	1.374 (0.808,2.338)	0.241
Hypertension	1.016 (0.804,1.285)	0.892	0.950 (0,715,1.261)	0.722	1.013 (0.784,1.309)	0.921
Urinary iodine concentration	1.000 (0.999,1.001)	0.955	1.000 (0.999,1.001)	0.679	1.000 (0.999,1.001)	0.845
Multiple logistic analysis						
Sex	–	–	–	–	3.284 (2.368,4.553)	< 0.001
Age	–	–	–	–	0.845 (0.594,1.203)	0.350
Smoking	–	–	–	–	0.671 (0.420,1.070)	0.094
Obesity	–	–	–	–	1.504 (1.077,2.101)	0.017

Univariate logistic regression, when adjusted for sex (male and female), age (<65y and ≥ 65y), smoking status (non-smoking and smoking), obesity (no and yes), diabetes mellitus (no and yes), hypertension (no and yes), urinary iodine concentration (continuous variable) was used for identify the associated risk factors for positive thyroid autoantibodies (model 1), positive TPO-Ab (model 2) and Tg-Ab (model 3), respectively. Factors that differed in the univariate logistic analysis were further analysed in the multiple logistic analysis. *P* < 0.05 was considered significant

*TPO-Ab* Thyroid peroxidase antibody, *Tg-Ab* Thyroglobulin antibody

## Discussion

Subclinical hypothyroidism is associated with subtle proatherogenic abnormalities since childhood. Both obesity and subclinical hypothyroidism have adverse influences on the metabolism of the human body [13, 14]. Hence, identifying the relationship between obesity and SCH is important in controlling risk factors and improving prognosis. This current study found a higher proportion of subclinical hypothyroidism in obese than nonobese subjects among those with positive thyroid autoantibodies. Moreover, obesity was significantly associated with subclinical hypothyroidism only in the presence of thyroid autoantibodies. Tg-Ab was more common in obese subjects, and there was a significant association between obesity and Tg-Ab.

The results of the current study are in line with the Tehran Thyroid Study, which reported a higher prevalence of subclinical hypothyroidism in obese subjects than in normal-weight subjects [7]. Similarly, a higher prevalence of subclinical hypothyroidism in overweight/obese subjects than in lean subjects was found in a Danish survey [8]. However, an Indian study reported no significant difference in the prevalence of subclinical hypothyroidism between overweight and lean PCOS patients [15]. The different BMIs in the obesity definition and the different TSHs in the subclinical hypothyroidism definition may partially explain this discrepancy. Moreover, interestingly, a higher proportion of subclinical hypothyroidism in obese individuals was only observed in the presence of thyroid autoantibodies in our study. In addition, a significant association between obesity and subclinical hypothyroidism was observed only in the presence of positive thyroid autoantibodies. Our findings are in agreement with those from another study conducted in China indicating that obesity was associated with hyperthyrotropinemia only in individuals with thyroid autoimmunity [16]. The presence of thyroid autoantibodies may mediate the association between obesity and subclinical hypothyroidism. Examining subclinical hypothyroidism in obese subjects with positive thyroid autoantibodies may benefit patients.

The present study further explored the relationship between obesity and TPO-Ab or Tg-Ab. The results showed that obese subjects had a higher Tg-Ab positivity rate than nonobese subjects but not a higher TPO-Ab positivity rate. When adjusting for sex, age and smoking, obesity was still associated with Tg-Ab positivity. Marzullo P and his colleagues reported similar results as ours [17]. However, they also observed a significantly higher rate of TPO-Ab positivity in obese subjects. Another cohort study failed to find an association between obesity and the TPO-Ab positivity [18]. Thus, the association between obesity and TPO-Ab or Tg-Ab positivity remains inconsistent. One experimental study found that Tg-Ab and Tg were deposited in the thyroid gland of obese strain chickens, and Tg-Ab was generated more rapidly in an autoimmune thyroiditis rat model than TPO-Ab [19, 20]. Obesity may be related to both TPO-Ab and Tg-Ab, but the generation of Tg-Ab may occur earlier, followed by TPO-Ab. The different courses of obesity may explain the inconsistent results among the above studies. Further study is needed.

Several studies have reported positive correlations between the TSH concentration and BMI [21–23]. Our results are in line with these reports. However, other studies found inconsistent results [24, 25]. This discrepancy may be due to differences in iodine nutrition and smoking statuses. A recent study found that BMI was a factor for monitoring iodine nutritional status [26]. Iodine nutrition is closely related to the TSH concentration. In addition, our study found that smoking was negatively related to the TSH concentration, similar to reports conducted in Italy and Denmark [27, 28]. Lower levels of serum TSH were observed among smokers. The protective effect mediated by the competitive inhibition of iodine uptake by thiocyanate, a degradation product of cyanide in tobacco, may explain this result [29]. Additionally, the relationship between TSH and high body mass differs between smokers and never-smokers [22].

This study was a cross-sectional survey. Hence, the relationship among obesity, subclinical hypothyroidism and thyroid autoantibodies should be explored further in prospective cohort studies to confirm. In addition, serum concentrations of FT4 were examined only in subjects with abnormal TSH concentrations, and associations between obesity and FT4 were not analysed in this study.

## Conclusions

In summary, this study found a significantly higher proportion of subclinical hypothyroidism in obese subjects than in nonobese subjects in the presence of thyroid autoantibodies. Moreover, obesity was significantly associated with increased odds of subclinical hypothyroidism among these subjects after adjusting for confounding factors. Tg-Ab was much more common in obese subjects, and obesity was related to Tg-Ab positivity. Our results suggested that thyroid autoantibodies might mediate the relationship between obesity and subclinical hypothyroidism. Assessing subclinical hypothyroidism in obese subjects with positive thyroid autoantibodies may benefit patients and is recommended.

## Data Availability

The datasets analysed during the current study are available from the corresponding author on reasonable request.

## Abbreviations

BMI: Body mass index (BMI)
CI: Confidence interval
FT3: Free triiodothyronine
FT4: Free thyroxine
OR: Odds ratio
PCOS: Polycystic ovarian syndrome
SCH: Subclinical hypothyroidism
SD: Standard deviations
TPO-Ab: Thyroid peroxidase antibody
TSH: Thyroid stimulating hormone
Tg-Ab: Thyroglobulin antibody
